# Association of obesity, triglyceride-glucose and its derivatives index with risk of hyperuricemia among college students in Qingdao, China

**DOI:** 10.3389/fendo.2022.1001844

**Published:** 2022-10-06

**Authors:** Shizhe Zhou, Yajie Yu, Zengxiao Zhang, Lidan Ma, Can Wang, Min Yang, Xiaoyu Cheng, Tian Liu, Ruixia Sun, Ying Chen

**Affiliations:** ^1^ Department of Endocrinology and Metabolism, The Affiliated Hospital of Qingdao University, Qingdao, China; ^2^ Department of Medicine, Qingdao University, Qingdao, China

**Keywords:** hyperuricemia, triglyceride-glucose, obesity, serum uric acid, college students

## Abstract

**Objective:**

To analyze and compare the associations of hyperuricemia (HUA) with obesity, triglyceride-glucose (TyG), and its derivatives in college students. To provide early guidance on risk predictors of HUA in college students.

**Methods:**

This study was a cross-sectional survey including 23,411 participants (age: 17-20 years). Investigators conducted face-to-face interview surveys and physical examinations. Automated biochemical methods were used to detect biochemical indicators such as serum uric acid (UA). Calculation of obesity, TyG, and their derivatives indices were performed. Logistic regression was used to analyze the relationship between different indexes and hyperuricemia. OR value and 95% CI were also calculated. ROC curve was used for assessing the predictive ability of different indices of hyperuricemia.

**Results:**

After adjusting for age, SBP, DBP, ALT, AST, TC, BUN, and CREA, multivariate logistic regression showed that the OR value of LAP in the obesity index was higher, especially in women (male OR: 4.347, 95%CI: 3.807, 4.964; female OR: 4.672, 95%CI: 3.800, 5.744). The other three quartiles of TyG derivatives were highly associated with hyperuricemia in men and women compared with the top quartile (all P< 0.05). The risk of hyperuricemia increased with an increase in quartiles. For college students, all indicators could distinguish the presence of hyperuricemia. For men, the area under the curve (AUC) of TyG-WC was the largest (AUC: 0.694; 95%CI: 0.684-0.704; P<0.05), according to the Maximum Youden index 0.290 with cut point value 477.853. In women, TyG-BMI showed a maximum AUC value of 0.702 (95%CI: 0.685-0.719; P<0.05), according to the maximum Youden index of 0.317 with cut point value 132.446. The TyG-WC, TyG-WHtR, TyG-LAP, and LAP indices also had relatively high AUC.

**Conclusion:**

In clinical practice, LAP, TYG, and their related derivatives may be used as sensitive indicators for HUA prediction in college students.

## Introduction

Hyperuricemia (HUA) is a metabolic disease caused by either increased production or insufficient excretion of uric acid from the body. According to the National Health and Nutrition Examination Survey of the United States, about 21% of American adults have HUA (1). The age-standardized prevalence of HUA is about 11.4% (2). A national survey showed that the overall prevalence of hyperuricemia in the Chinese adult population was 11.1% in 2015-16, which increased to 14.0% in 2018-19 (3). Of note, among young people aged 18-29, the prevalence of hyperuricemia increased from 13.4 to 18.0% in three years (3). The rise in prevalence of HUA globally has gradually become a public health burden.

Previous studies have demonstrated that obesity and insulin resistance (IR) is associated with HUA (4, 5). A positive correlation exists between visceral fat deposition and increased uric acid production (6). However, visceral fat requires a diagnostic imaging technique, which is expensive. So it is crucial to find simple and effective surrogate markers for assessing visceral fat. Moreover, traditional obesity indicators such as body mass index (BMI), waist circumference (WC), and waist-height ratio (WHtR) cannot differentiate between visceral and subcutaneous fat. Some newly proposed obesity-related indices, such as visceral adiposity index (VAI), lipid accumulation product (LAP) index, plasma atherosclerosis index (AIP), cardiometabolic index (CMI), body shape index (ABSI), and body roundness index (BRI) provides an idea for predicting the occurrence of related metabolic diseases. There is a close relationship between insulin resistance (IR) and glucose with lipid metabolism. The triglyceride-glucose index (TyG) has been proposed as a simple substitute for IR (7). Studies have shown the effectiveness of TyG in assessing IR (8). In addition, its efficiency in assessing IR may be improved when TyG is combined with some other obesity indicators such as body mass index (BMI), waist circumference (WC), and waist circumference ratio (WTHR) (8–10). Nonetheless, IR can lead to the occurrence and progression of HUA, and timely identification and intervention of IR may be beneficial in preventing HUA and its related diseases.

Although obesity indicators and insulin resistance index can predict the risk of HUA (11, 12), the more suitable indicator for predicting the risk of HUA in Chinese college students has yet to be concluded. Therefore, this study used a cross-sectional survey to analyze and compare the three indices of visceral adiposity (LAP index, ABSI, and BRI), general adiposity index (BMI), abdominal adiposity index (WC and WHtR), with the TyG index and its derivatives to predict the risk of HUA. In addition, to identify more appropriate risk predictors of HUA in the college student population for providing a basis for early prevention of HUA.

## Method

### Research subjects and methods:

A total of 23,411 participants (age: 17-20 years old) from the physical examination population of Qingdao University were included from September 2017 to October 2019. Exclusion criteria: (1) chronic kidney disease or renal impairment, (2) long-term use of uric acid-lowering drugs, (3) malignant tumors and autoimmune diseases. Investigators conducted interviews for the gender, age, history, family history of hypertension, etc. In addition, height, weight, blood pressure, waist, and hip circumference were measured. Besides alanine aminotransferase (ALT), aspartate aminotransferase (AST), triglyceride (TG), total cholesterol (TC), fasting blood glucose (FPG), urea nitrogen (BUN), creatinine (Cre), uric acid (UA) and other biochemical indicators were analyzed.

2.2 According to the definition from “Guidelines for Diagnosis and Treatment of Hyperuricemia and Gout in China (2019)”, two measurements on different days, UA > 420 μmol/l is considered hyperuricemia (13). Therefore, based on the previous studies (12, 14), it was calculated using the following formula:


$$\text{BMI}=\text{weight (kg})/{\text{height}}^{2}({\text{m}}^{2}),$$

WHtR=waist circumference (cm)/height(cm),

LAP index (male)=TG(mmol/l)/(WC[cm]−58),

LAP index (female)=TG(mmol/l)/(WC[cm]−65),

TyG index=ln(TG[mg/dl]×GLU[mg/dl]/2),

$$\text{ABSI}=\text{WC(cm)}/{\left(\text{height}[\text{cm}]\right)}^{1/2}\times {\left(\text{BMI}2\right)}^{1/2},$$

$$\text{BRI}=364.2-365.5\times {\left[1-(\text{WC}/2\Pi )/(0.5\times \text{height})2\right]}^{1/2},$$

TyG−BMI=TyG×BMI,

TyG−WC=TyG×WC,

TyG−WHtR=TyG×WHtR,

TyG−LAP=TyG×LAP.

### Statistical analysis

Normally distributed measurement data were expressed as mean ± standard deviation ( $\bar{\text{x}}\pm \text{s}$ ), and the student t-test was used to compare groups. While the non-normally distributed measurement data was expressed as M (P25, P75), and comparison between groups was assessed using the Rank sum test. The count data were expressed as frequency (%), and the Kruskal-Wallis H test was used to compare groups. Logistic regression analysis was used to analyze the relationship between different indicators and hyperuricemia; OR value and 95% CI were calculated. ROC curve analysis was used to show the predictive ability of different indicators for hyperuricemia. The Delong test was used to compare the AUC values of multiple groups. SPSS26.0, R software was used for statistical analysis and GraphPad Prism 8.0 for the generation of graphs. A two-sided P<0.05 was considered statistically significant.

## Results

### Baseline data of the study population and comparison of clinical characteristics between the hyperuricemia and the non-hyperuricemia group

A total of 23,411 participants, including 11,177 males (47.74%) and 12,234 females (52.26%) aged 17-20 years, with an average age of 18.28 ± 0.64 years, were included in the study. The basic data characteristics of all participants are shown in **Table 1**.

**Table 1:** Comparison of baseline characteristics of study participants who developed hyperuricemia or not.

Characteristics	Total (n = 23411)	Non-hyperuricemia (n = 16667)	Hyperuricemia (n = 6744)	c^2^/t/z	P-value
Males,n (%)	11177 (47.74%)	5572 (33.43%)	5605 (83.11%)	4749.601	< 0.001
Age (years)	18.28 ± 0.64	18.28 ± 0.64	18.29 ± 0.63	-1.273	0.203
Height (cm)	169.06 ± 8.37	167.24 ± 8.02	173.57 ± 7.47	-57.471	< 0.001
Weight (kg)	61.90 ± 13.18	58.18 ± 10.23	71.10 ± 15.00	-64.920	< 0.001
SBP (mmHg)	111.63 ± 12.24	109.26 ± 11.55	117.48 ± 11.93	-48.170	< 0.001
DBP (mmHg)	70.23 ± 7.85	69.59 ± 7.60	71.82 ± 8.25	-19.157	< 0.001
SUA (μmol/l)	374.25 ± 102.02	323.28 ± 58.18	500.23 ± 73.97	-175.689	< 0.001
FPG (mmol/l)	4.29 ± 0.64	4.24 ± 0.62	4.41 ± 0.66	-18.640	< 0.001
TC (mmol/l)	3.94 ± 0.72	3.90 ± 0.70	4.06 ± 0.77	-15.473	< 0.001
TG (mmol/l)	0.69 (0.57-0.87)	0.67 (0.56-0.83)	0.76 (0.61,0.97)	-24.123	< 0.001
BUN (mmol/l)	4.50 ± 1.23	4.32 ± 1.18	4.93 ± 1.26	-33.799	< 0.001
Cre (mmol/l)	76.25 ± 16.24	72.14 ± 15.21	86.40 ± 14.10	-68.499	< 0.001
ALT (mmol/l)	14.00 (10.00-20.00)	13.00 (10.00,17.00)	18.00 (13.00,28.00)	-30.016	< 0.001
AST (mmol/l)	19.00 (17.00-23.00)	19.00 (16.00,22.00)	21.00 (18.00,26.00)	-23.628	< 0.001
TyG index	6.19 ± 0.40	6.14 ± 0.38	6.31 ± 0.42	-28.933	< 0.001
BMI (kg/m2)	21.55 ± 3.66	20.75 ± 2.97	23.54 ± 4.39	-47.938	< 0.001
WC (cm)	73.93 ± 10.28	71.30 ± 8.21	80.43 ± 11.86	-57.783	< 0.001
WHtR	0.43 ± 0.06	0.43 ± 0.05	0.46 ± 0.07	-41.616	< 0.001
LAP index	7.32 (3.72,13.20)	6.45 (3.36-11.01)	10.85 (5.13,20.60)	-35.057	< 0.001
ABSI	0.74 ± 0.05	0.73 ± 0.05	0.75 ± 0.05	-19.526	< 0.001
BRI	2.32 ± 0.99	2.13 ± 0.81	2.78 ± 1.23	-40.445	< 0.001
TyG-BMI	133.78 ± 26.94	126.68 ± 20.20	146.57 ± 31.48	-20.877	< 0.001
TyG-WC	458.65 ± 78.57	429.14 ± 56.13	480.87 ± 82.49	-20.653	< 0.001
TyG-WHtR	2.71 ± 0.44	2.63 ± 0.35	2.94 ± 0.50	-20.559	< 0.001
TyG-LAP	44.77 (22.20,82.96)	39.13 (20.06,68.60)	67.65 (31.19,132.03)	-16.004	< 0.001

SBP, systolic blood pressure; DBP, diastolic blood pressure; SUA, serum uric acid; FPG, fasting plasma glucose; TC, total cholesterol; TGs, triglycerides; BUN, blood urea nitrogen; Cre, creatinine;ALT, alanine aminotransferase;AST, aspartate aminotransferase;TyG index, triglyceride glucose index; BMI, body mass index; WC, waist circumference;WHtR, waist-to-height ratio; LAP index, lipid accumulation product index; ABSI, a body shape index; BRI, body roundness index.

**Figure 1:**
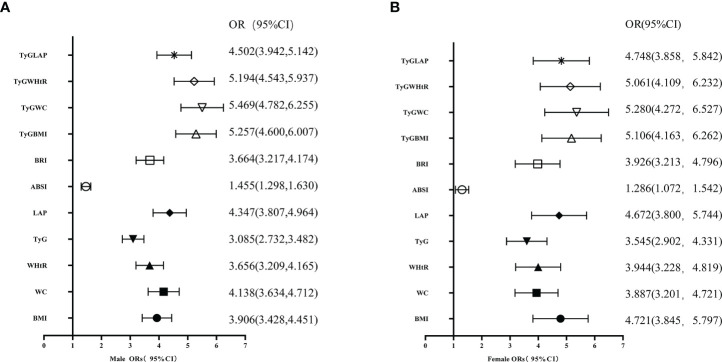
**(A)** Multivariate logistic regression OR values adjusted for age, SBP, and DBP,BUN, Cre, ALT,AST,TC, and LDL-C in males. **(B)** Multivariate logistic regression OR values adjusted for age, SBP, and DBP,BUN, Cre, ALT,AST,TC, and LDL-C in females.OR and 95% confidence interval for the incidence of hyperuricemia in the high versus low quartile of obesity markers, TyG and its derivatives. The multivariate adjusted OR were adjusted for age, SBP, DBP, BUN, Cre, ALT,AST,TC, and LDL-C; CI, confidence intervals; TyG, triglyceride and glucose index; BMI, body mass index; WC, waist circumference; WHtR, waist-to-height ratio; AIP, atherogenic index of plasma; CMI, cardiometabolic index; VAI, visceral adiposity index; LAP index, lipid accumulation product index; ABSI, a body shape index; BRI, body roundness index. TyGBMI,TyG-BMI =TyG×BMI; TyGWC, TyG-WC=TyG×WC; TyGWHtR ,TyG-WHtR=TyG×WHtR; TyG-LAP,TyG-LAP=TyG×LAP. =TyG×BMI.

Based on the presence or absence of hyperuricemia, the study population was divided into the non-hyperuricemia group (NHUA group) and the hyperuricemia group (HUA group). No significant difference was found in age between the two groups (P>0.05). However, compared with the NHUA group, the HUA group has significantly increased height, weight, systolic blood pressure (SBP), diastolic blood pressure (DBP), ALT, AST, TG, TC, FPG, BUN, CREA, TyG index, BMI, WC, WHtR, LAP index, ABSI, BRI, TyG-BMI, TyG-WC, TyG-WHtR, and TyG-LAP (P<0.05) (**Figure 1**).

### Analysis of risk factors for hyperuricemia

With hyperuricemia as the dependent variable, TyG index, BMI, WC, WHtR, LAP index, ABSI, BRI, TyG-BMI, TyG-WC, TyG-WHtR, and TyG-LAP quartiles were used as independent groups. Multivariate logistic regression calculated the OR value and 95% CI. Following the model 3 adjustment for age, SBP, DBP, ALT, AST, TC, BUN, and CREA, the OR value of LAP in the obesity index was higher, especially in men, compared with the top quartile. In men, the other three quartiles of TyG derivatives were highly associated with hyperuricemia (all P<0.05), and the risk of developing hyperuricemia increased with increasing quartiles of TyG derivatives.Similar results were obtained in women. The results are shown in **Table 2** and **Table 3**. Model 3, adjusted for male or female variables, revealed that the OR value of TyG derivatives in the bottom quartile was higher than that of the single indicator compared with the top quartile. The results are shown in **Tables 3** and **4**.

**Table 2:** Multivariate logistic regression of different indices for HUA (males).

Variables	Quartile 1	Quartile 2		Quartile 3		Quartile 4	
		OR (95% CI)	P-value	OR (95% CI)	P-value	OR (95% CI)	P-value
TyG index	≤5.9576	5.9576-6.2011		6.2011-6.4671		≥6.4671	
Model 1	Reference	1.277 (1.148,1.422)	< 0.001	1.868 (1.679,2.078)	< 0.001	3.243 (2.906,3.618)	< 0.001
Model 2	Reference	1.283 (1.152,1.429)	< 0.001	1.848 (1.660,2.058)	< 0.001	3.110 (2.784,3.475)	< 0.001
Model 3	Reference	1.479 (1.319,1.658)	< 0.001	2.071 (1.846,2.324)	< 0.001	3.085 (2.732,3.482)	< 0.001
BMI	≤19.3792	19.3792-21.5619		21.5619-24.5351		≥24.5351	
Model 1	Reference	1.351 (1.212,1.507)	< 0.001	2.284 (2.050,2.544)	< 0.001	5.287 (4.716,5.927)	< 0.001
Model 2	Reference	1.312 (1.176,1.465)	< 0.001	2.166 (1.940,2.419)	< 0.001	4.834 (4.285,5.453)	< 0.001
Model 3	Reference	1.194 (1.065,1.338)	< 0.001	1.889 (1.682,2.121)	< 0.001	3.906 (3.428,4.451)	< 0.001
WC	≤70	70-76		76-84		≥84	
Model 1	Reference	1.475 (1.325,1.641)	< 0.001	2.230 (2.004,2.481)	< 0.001	5.638 (5.033,6.315)	< 0.001
Model 2	Reference	1.436 (1.290,1.599)	< 0.001	2.124 (1.904,2.369)	< 0.001	5.199 (4.613,5.861)	< 0.001
Model 3	Reference	1.270 (1.136,1.421)	< 0.001	1.788 (1.594,2.005)	< 0.001	4.138 (3.634,4.712)	< 0.001
WHtR	≤0.3988	0.3988-0.4332		0.4332-0.4800		≥0.4800	
Model 1	Reference	1.357 (1.217,1.512)	< 0.001	2.102 (1.887,2.343)	< 0.001	5.008 (4.467,5.614)	< 0.001
Model 2	Reference	1.316 (1.180,1.468)	< 0.001	1.983 (1.776,2.214)	< 0.001	4.526 (4.017,5.100)	< 0.001
Model 3	Reference	1.202 (1.073,1.347)	0.002	1.718 (1.530,1.929)	< 0.001	3.656 (3.209,4.165)	< 0.001
LAP index	≤2.9600	2.9600-7.3200		7.3200-15.0350		≥15.0350	
Model 1	Reference	1.475 (1.322,1.645)	< 0.001	2.339 (2.098,2.608)	< 0.001	5.750 (5.122,6.454)	< 0.001
Model 2	Reference	1.424 (1.275,1.590)	< 0.001	2.209 (1.977,2.469)	< 0.001	5.272 (4.671,5.951)	< 0.001
Model 3	Reference	1.315 (1.173,1.474)	< 0.001	1.934 (1.721,2.173)	< 0.001	4.347 (3.807,4.964)	< 0.001
ABSI	≤0.7182	0.7182-0.7408		0.7408-0.7659		≥0.7659	
Model 1	Reference	1.211 (1.090,1.345)	< 0.001	1.343 (1.209,1.492)	< 0.001	1.823 (1.639,2.027)	< 0.001
Model 2	Reference	1.203 (1.082,1.338)	0.001	1.295 (1.165,1.441)	< 0.001	1.714 (1.539,1.908)	< 0.001
Model 3	Reference	1.112 (0.995,1.243)	0.061	1.138 (1.018,1.273)	0.023	1.455 (1.298,1.630)	< 0.001
BRI	≤1.6575	1.6575-2.1908		2.1908-2.9914		≥2.9914	
Model 1	Reference	1.365 (1.224,1.521)	< 0.001	2.106 (1.890,2.346)	< 0.001	5.016 (4.475,5.622)	< 0.001
Model 2	Reference	1.324 (1.187,1.476)	< 0.001	1.986 (1.780,2.217)	< 0.001	4.534 (4.025,5.108)	< 0.001
Model 3	Reference	1.209 (1.079,1.354)	0.001	1.722 (1.534,1.933)	< 0.001	3.664 (3.217,4.174)	< 0.001
TyG-BMI	≤117.7455	117.7455-133.1496		133.1496-154.8333		≥154.8333	
Model 1	Reference	1.504 (1.348,1.679)	< 0.001	2.547 (2.283,2.841)	< 0.001	6.440 (5.730,7.238)	< 0.001
Model 2	Reference	1.474 (1.320,1.647)	< 0.001	2.439 (2.182,2.725)	< 0.001	5.986 (5.298,6.763)	< 0.001
Model 3	Reference	1.429 (1.273,1.603)	< 0.001	2.279 (2.028,2.562)	< 0.001	5.257 (4.600,6.007)	< 0.001
TyG-WC	≤422.9615	422.9615-468.0405		468.0405-530.9639		≥530.9639	
Model 1	Reference	1.507 (1.350,1.682)	< 0.001	2.485 (2.227,2.771)	< 0.001	6.574 (5.846,7.393)	< 0.001
Model 2	Reference	1.476 (1.321,1.648)	< 0.001	2.381 (2.131,2.661)	< 0.001	6.109 (5.407,6.904)	< 0.001
Model 3	Reference	1.464 (1.305,1.643)	< 0.001	2.229 (1.984,2.506)	< 0.001	5.469 (4.782,6.255)	< 0.001
TyG-WHtR	≤2.4181	2.4181-2.6781		2.6781-3.0360		≥3.0360	
Model 1	Reference	1.520 (1.362,1.696)	< 0.001	2.429 (2.178,2.709)	< 0.001	6.204 (5.522,6.971)	< 0.001
Model 2	Reference	1.487 (1.332,1.660)	< 0.001	2.333 (2.090,2.605)	< 0.001	5.697 (5.050,6.428)	< 0.001
Model 3	Reference	1.475 (1.315,1.654)	< 0.001	2.234 (1.989,2.510)	< 0.001	5.194 (4.543,5.937)	< 0.001
TyG-LAP	≤17.6338	17.6338-44.8022		44.8022-94.9628		≥94.9628	
Model 1	Reference	1.503 (1.348,1.677)	< 0.001	2.393 (2.146,2.668)	< 0.001	5.878 (5.235,6.600)	< 0.001
Model 2	Reference	1.454 (1.302,1.623)	< 0.001	2.265 (2.027,2.531)	< 0.001	5.394 (4.779,6.089)	< 0.001
Model 3	Reference	1.345 (1.199,1.508)	< 0.001	2.019 (1.797,2.269)	< 0.001	4.502 (3.942,5.142)	< 0.001

Model 1: unadjusted; model 2: adjusted for age, SBP, and DBP; model 3: adjusted for all variables in model 2 plus BUN, Cre, ALT,AST, and TC. TyG index, triglyceride glucose index; BMI,body mass index; WC, waist circumference; WHtR, waist-to-height ratio; AIP, atherogenic index of plasma; CMI, cardiometabolic index; VAI, visceral adiposity index; LAP index, lipid accumulation product index; ABSI, a body shape index; BRI, body roundness index.

**Table 3:** Multivariate logistic regression of different indices for HUA (females).

Variables	Quartile 1	Quartile 2		Quartile 3		Quartile 4	
		OR (95% CI)	P-value	OR (95% CI)	P-value	OR (95% CI)	P-value
TyG index	≤5.9261	5.9261-6.1588		6.1588-6.3992		≥6.3992	
Model 1	Reference	1.334 (1.081,1.647)	0.007	2.020 (1.660,2.459)	< 0.001	3.072 (2.549,3.703)	< 0.001
Model 2	Reference	1.327 (1.075,1.639)	0.009	2.003 (1.644,2.440)	< 0.001	2.997 (2.483,3.616)	< 0.001
Model 3	Reference	1.695 (1.364,2.107)	< 0.001	2.547 (2.072,3.131)	< 0.001	3.545 (2.902,4.331)	< 0.001
BMI	≤18.7500	18.7500-20.3125		20.3125-22.2656		≥22.2656	
Model 1	Reference	1.390 (1.105,1.750)	0.005	1.954 (1.573,2.428)	< 0.001	5.177 (4.256,6.297)	< 0.001
Model 2	Reference	1.378 (1.094,1.735)	0.006	1.920 (1.544,2.388)	< 0.001	4.732 (3.875,5.777)	< 0.001
Model 3	Reference	1.425 (1.129,1.800)	0.003	1.955 (1.566,2.440)	< 0.001	4.721 (3.845,5.797)	< 0.001
WC	≤65	65-69		69-74		≥74	
Model 1	Reference	1.258 (1.005,1.576)	0.046	2.266 (1.848,2.778)	< 0.001	4.620 (3.836,5.564)	< 0.001
Model 2	Reference	1.253 (1.000,1.570)	0.050	2.204 (1.796,2.704)	< 0.001	4.213 (3.488,5.090)	< 0.001
Model 3	Reference	1.224 (0.973,1.538)	0.084	2.163 (1.757,2.662)	< 0.001	3.887 (3.201,4.721)	< 0.001
WHtR	≤0.3970	0.3970-0.4231		0.4231-0.4557		≥0.4557	
Model 1	Reference	1.212 (0.964,1.525)	0.100	2.096 (1.701,2.583)	< 0.001	4.508 (3.718,2.583)	< 0.001
Model 2	Reference	1.216 (0.966,1.530)	0.095	2.073 (1.681,2.556)	< 0.001	4.161 (3.424,5.057)	< 0.001
Model 3	Reference	1.231 (0.975,1.554)	0.081	2.071 (1.674,2.562)	< 0.001	3.944 (3.228,4.819)	< 0.001
LAP index	≤4.2270	4.2270-7.3200		7.3200-12.0375		≥12.0375	
Model 1	Reference	1.375 (1.089,1.737)	0.007	2.149 (1.730,2.670)	< 0.001	5.192 (4.257,6.331)	< 0.001
Model 2	Reference	1.356 (1.073,1.713)	0.011	2.077 (1.671,2.582)	< 0.001	4.730 (3.870,5.781)	< 0.001
Model 3	Reference	1.390 (1.098,1.761)	0.006	2.114 (1.695,2.637)	< 0.001	4.672 (3.800,5.744)	< 0.001
ABSI	≤0.6976	0.6976-0.7243		0.7243-0.7553		≥0.7553	
Model 1	Reference	1.316 (1.102,1.573)	0.003	1.230 (1.027,1.474)	0.024	1.375 (1.152,1.641)	< 0.001
Model 2	Reference	1.318 (1.102,1.577)	0.003	1.245 (1.039,1.493)	0.018	1.387 (1.161,1.657)	< 0.001
Model 3	Reference	1.282 (1.067,1.540)	0.008	1.156 (0.960,1.392)	0.127	1.286 (1.072,1.542)	0.007
BRI	≤1.6297	1.6297-2.0303		2.0303-2.5655		≥2.5655	
Model 1	Reference	1.210 (0.962,1.522)	0.103	2.101 (1.705,2.589)	< 0.001	4.483 (3.697,5.435)	< 0.001
Model 2	Reference	1.214 (0.965,1.527)	0.099	2.076 (1.683,2.560)	< 0.001	4.138 (3.406,5.029)	< 0.001
Model 3	Reference	1.230 (0.974,1.552)	0.082	2.075 (1.677,2.567)	< 0.001	3.926 (3.213,4.796)	< 0.001
TyG-BMI	≤113.4858	113.4858-124.7447		124.7447-138.7289		≥138.7289	
Model 1	Reference	1.195 (0.945,1.512)	0.136	1.886 (1.519,2.341)	< 0.001	5.263 (4.334,6.392)	< 0.001
Model 2	Reference	1.177 (0.930,1.488)	0.175	1.831 (1.474,2.275)	< 0.001	4.806 (3.945,5.854)	< 0.001
Model 3	Reference	1.315 (1.036,1.669)	0.024	2.020 (1.619,2.521)	< 0.001	5.106 (4.163,6.262)	< 0.001
TyG-WC	≤392.0616	392.0616-424.6261		424.6261-464.2593		≥464.2593	
Model 1	Reference	1.509 (1.192,1.912)	0.001	2.303 (1.846,2.873)	< 0.001	5.574 (4.548,6.830)	< 0.001
Model 2	Reference	1.491 (1.177,1.889)	0.001	2.233 (1.789,2.787)	< 0.001	5.107 (4.159,6.270)	< 0.001
Model 3	Reference	1.620 (1.274,2.059)	< 0.001	2.388 (1.906,2.992)	< 0.001	5.280 (4.272,6.527)	< 0.001
TyG-WHtR	≤2.3990	2.3990-2.6017		2.6017-2.8479		≥2.8479	
Model 1	Reference	1.391 (1.101,1.759)	0.006	2.253 (1.814,2.799)	< 0.001	5.205 (4.262,6.356)	< 0.001
Model 2	Reference	1.393 (1.101,1.761)	0.006	2.213 (1.781,2.751)	< 0.001	4.839 (3.957,5.918)	< 0.001
Model 3	Reference	1.499 (1.182,1.902)	0.001	2.369 (1.898,2.957)	< 0.001	5.061 (4.109,6.232)	< 0.001
TyG-LAP	≤25.1407	25.1407-44.6600		44.6600-75.4415		≥75.4415	
Model 1	Reference	1.406 (1.114,1.776)	0.004	2.154 (1.733,2.677)	< 0.001	5.223 (4.280,6.373)	< 0.001
Model 2	Reference	1.378 (1.091,1.740)	0.007	2.072 (1.666,2.577)	< 0.001	4.752 (3.886,5.812)	< 0.001
Model 3	Reference	1.423 (1.123,1.802)	0.003	2.145 (1.718,2.677)	< 0.001	4.748 (3.858,5.842)	< 0.001

Model 1: unadjusted; model 2: adjusted for age, SBP, and DBP; model 3: adjusted for all variables in model 2 plus BUN, Cre, ALT、AST、TC, and LDL-C. TyG index, triglyceride glucose index; BMI,body mass index; WC, waist circumference; WHtR, waist-to-height ratio; AIP, atherogenic index of plasma; CMI, cardiometabolic index; VAI, visceral adiposity index; LAP index, lipid accumulation product index; ABSI, a body shape index; BRI, body roundness index.

### ROC curve analysis of hyperuricemia

**Figure 2:**
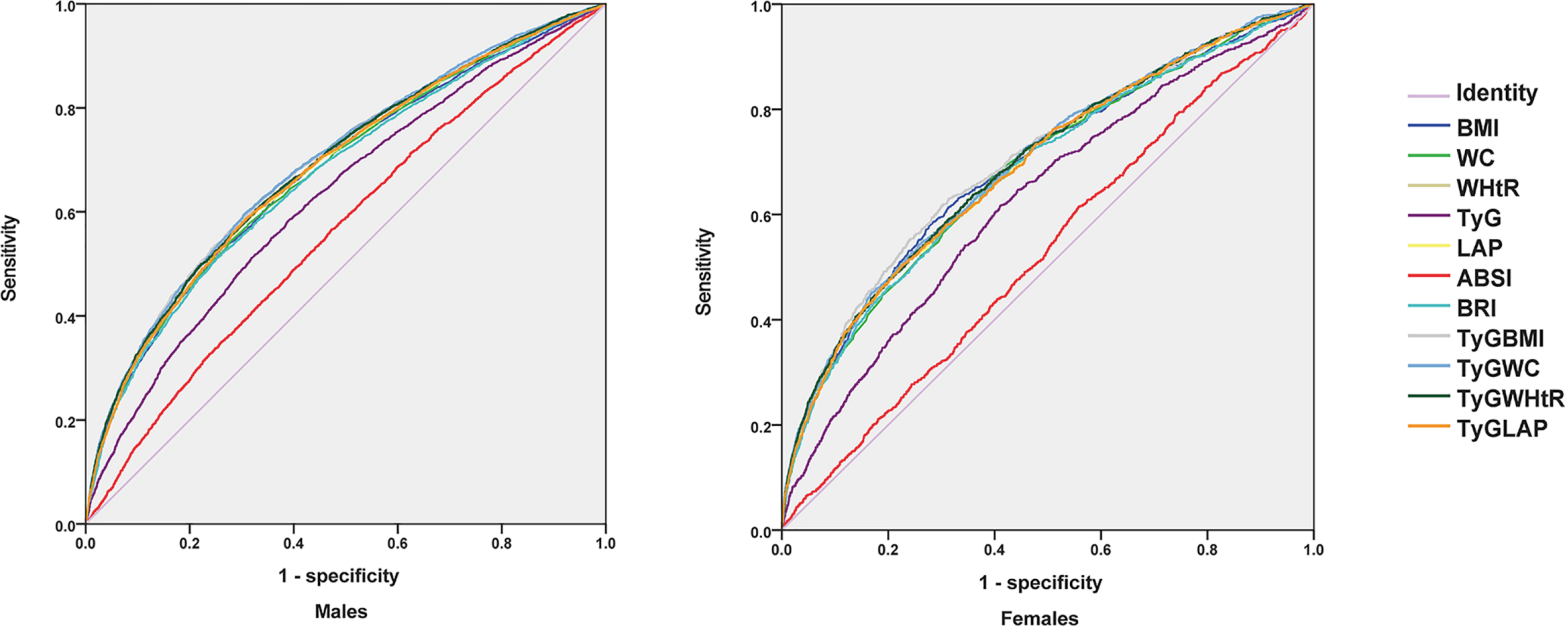
Receiver operating characteristic (ROC) curve analysis by sex. TyG index, triglyceride glucose index; BMI, body mass index; WC, waist circumference; WHtR, waist-to-height ratio; LAP index, lipid accumulation product index; ABSI, a body shape index; BRI, body roundness index.

The ROC curves of hyperuricemia under different indices are given in **Table 4** and **Figure 2**. All indicators could distinguish the presence of hyperuricemia. For men, the area under the curve (AUC) of TyG-WC was the largest (AUC: 0.694; 95%CI: 0.684-0.704; P<0.05). According to the maximum Youden index of 0.290, the cut point value was 477.853; TyG-BMI, TyG-WHtR, TyG-LAP, and LAP index also had relatively high AUC values. For women, TyG-BMI had a maximum AUC value of 0.702 (95% CI: 0.685-0.719; P< 0.05); according to a maximum Youden index of 0.317, the cut point value of 132.446, TyG-WC, TyG-WHtR, TyG- LAP and LAP index also had relatively high AUC values.

**Table 4:** Comparison of the ability of different indices to predict HUA.

Variable	AUC (95% CI)	Cut-off	Sensitivity	Specificity	Youden index	P-value
Males						
BMI	0.677 (0.667,0.687)	22.60	0.530	0.261	0.269	< 0.001
WC	0.678 (0.668,0.688)	78.3	0.541	0.276	0.265	< 0.001
WHtR	0.671 (0.661,0.681)	0.453	0.511	0.252	0.259	< 0.001
TyG index	0.629 (0.618,0.639)	6.215	0.581	0.388	0.193	< 0.001
LAP index	0.682 (0.672,0.692)	8.7	0.578	0.302	0.276	< 0.001
ABSI	0.564 (0.554,0.575)	0.739	0.565	0.472	0.093	< 0.001
BRI	0.671 (0.661,0.681)	2.516	0.511	0.252	0.259	< 0.001
TyG-BMI	0.692 (0.683,0.702)	137.458	0.585	0.297	0.288	< 0.001
TyG-WC	0.694 (0.684,0.704)	477.853	0.596	0.306	0.290	< 0.001
TyG-WHtR	0.688 (0.678,0.698)	2.848	0.500	0.222	0.278	< 0.001
TyG-LAP	0.683 (0.673,0.693)	52.593	0.588	0.309	0.279	< 0.001
Females						
BMI	0.690 (0.673,0.707)	21.72	0.582	0.281	0.301	< 0.001
WC	0.683 (0.666,0.701)	70.2	0.675	0.397	0.278	< 0.001
WHtR	0.681 (0.663,0.698)	0.444	0.571	0.304	0.267	< 0.001
TyG index	0.627 (0.610,0.644)	6.220	0.616	0.412	0.204	< 0.001
LAP index	0.690 (0.673,0.707)	11.878	0.509	0.231	0.279	< 0.001
ABSI	0.527 (0.510,0.545)	0.717	0.615	0.558	0.057	0.003
BRI	0.681 (0.663,0.698)	2.370	0.571	0.304	0.267	< 0.001
TyG-BMI	0.702 (0.685,0.719)	132.446	0.631	0.314	0.317	< 0.001
TyG-WC	0.696 (0.679,0.713)	457.958	0.542	0.258	0.285	< 0.001
TyG-WHtR	0.694 (0.677,0.711)	2.707	0.627	0.348	0.278	< 0.001
TyG-LAP	0.689 (0.672,0.706)	74.819	0.505	0.228	0.278	< 0.001

TyG index, triglyceride glucose index; BMI, body mass index; WC, waist circumference; WHtR, waist-to-height ratio; LAP index, lipid accumulation product index; ABSI, a body shape index; BRI, body roundness index.

## Discussion

### Key findings

With lifestyle changes, and the presence of the obesity epidemic, hyperuricemia (HUA) has become a global health problem. Two studies on the Chinese population reported a 12.1% and 15.6% incidence of HUA, respectively (15, 16), while the trend was more towards the younger population. Another Japanese-based study observed an incidence of HUA of 31.7 per 1000 persons/year (17). In this study, the detection rate of hyperuricemia was 28.81%, which was significantly higher than in previous epidemiological surveys in all age groups. HUA not only causes gout and chronic kidney disease but also play an important role in the development of cardiovascular diseases such as diabetes and hypertension (18, 19). As a young group of college students, the prevention and management of HUA deserve specific attention. Thus finding an HUA-sensitive risk screening index is very crucial. By analyzing the associations of obesity index, TyG index, and its derivatives with hyperuricemia in college students, it was found that TyG derivatives were more strongly associated with the risk of hyperuricemia than obesity index or TyG index.

### HUA and obesity indicators

Previous studies have demonstrated that BMI, WC, and WHTR are associated with HUA (20–22), and similar results were obtained in this study. BMI reflects the degree of obesity, while WC and WHTR reflect abdominal obesity. However, they cannot differentiate between subcutaneous and visceral fat. The visceral fat accumulation is more likely to lead to uric acid metabolism disorder than subcutaneous fat (6). ABSI and BRI are newly proposed obesity indicators that reflect the fat distribution. Previous studies have demonstrated a close relationship between them and HUA (23, 24), and similar results were observed in the present study. LAP is a new indicator for evaluating obesity calculated from WC and fasting TG levels. TG levels are closely related to visceral fat distribution (4), and it is closely related to the occurrence of HUA in both men and women. With the increase of LAP, the risk of HUA in women is higher; given the higher AUC, LAP showed a relatively higher HUA than other indicators of obesity Recognition ability, with cutoff values ​​of 8.7 and 11.878 for men and women, respectively. The sensitivity of the ROC curve was higher in women than in men. Studies from different populations reported similar predictive power. Of note, estrogen (E2) regulates the quality of adipose tissue, and there were differences in the content of fat distribution in different parts and genders (25). All women in this study were non-menopausal. Therefore, their estrogen levels were mainly regulated by gonadotropins released by the hypothalamus. The elevated blood uric acid levels may affect hypothalamic hormone secretion and lead to decreased gonadotropin production, resulting in decreased testosterone and E2 production (26). Decreased E2 redistributes fat in women (27, 28), resulting in increased abdominal fat and visceral fat. However, it has less effect on fat redistribution in men. This may explain the more sensitivity of female LAPs to HUA recognition found in this study.

### HUA and TyG-related indicators

Previous studies have demonstrated the close relationship between HUA and IR (29, 30). The gold standard for IR assessment is the hyperinsulinemic-euglycemic clamp (HEC) (31). However, due to its invasiveness, complexity, and time-consuming, HEC is difficult to apply in clinical practice. Moreover, the steady-state model assessment of the IR (HOMA-IR) index is much simpler than HEC (32). Besides, issues such as cost and reproducibility need to be considered for insulin testing. TyG was proposed by Simental-Mendia et al. (7) as a simple, practical, and usable surrogate marker for identifying insulin resistance. Several studies have shown that TyG maintains a good agreement with HEC and HOMA-IR (33). Therefore, in clinical practice, TyG provides more options for assessing IR and preventing IR-related diseases. Existing studies have demonstrated that TyG indicators are associated with diabetes, hypertension, non-alcoholic fatty liver disease, and atherosclerosis (34–36). In addition, TyG is significantly associated with hyperuricemia (37). Our findings concur with the above results, showing a stable positive correlation between the TyG index and hyperuricemia. Obesity plays a crucial role in the pathophysiology of IR. Thus TyG combined with obesity indicators should theoretically enhance the effect of TyG. Multiple studies have demonstrated the superiority of TyG combined with obesity indicators (9, 10). According to previous studies, women with hyperuricemia have greater regression coefficients and odds ratios with TyG and its derivatives than men, which is similar to the present findings. The results can be explained by gender differences in fat distribution, glucose, lipid, and urate metabolism (38). It is worth noting that TyG-WC is the index with the largest odds ratio with hyperuricemia in women. In men, TyG-WHtR is the index with the largest odds ratio with hyperuricemia. WHtR is the index with the largest odds ratio for hyperuricemia (11), and results may vary based on the different populations. Overall, in men and women, the predictive effect between TyG derivatives and hyperuricemia was better than that of TyG and a single indicator of obesity. Of note, TyG-BMI was a more sensitive indicator in women and men. The highest index was TyG-WHtR, and further studies are required to explore the reason.

The main advantage of this cross-sectional study was the large sample size, which provided more statistical power.

However, this study had some limitations. First, in the absence of follow-up observations, diet and exercise can greatly affect the serum uric acid levels leading to biasness. Second, lack of adjustment for diet, plasma insulin levels, and family history may have also added bias to the findings. Third, the age of our study population was limited. Therefore, the results should be further validated in other ethnic and age groups. Fourth, this study was derived from the results of a physical examination. Thus it lacks data related to high-density lipoprotein cholesterol. Moreover, the evaluation of obesity indicators was not comprehensive. In addition, more elaborative data is needed to compare other indicators of obesity.

## Conclusion

In conclusion, LAP, as an obesity indicator, can reflect the accumulation of visceral fat. It was significantly associated with the risk of HUA and is more sensitive for identifying hyperuricemia than traditional obesity indicators. TyG, a non-insulin-based insulin resistance index, combined with an indicator of obesity, was significantly associated with HUA risk in both men and women. Overall, LAP, TYG, and their related derivatives can potentially be used as sensitive indicators for HUA prediction in clinical practices. However, further studies with longer observation periods are required to validate these findings.

## Data availability statement

The raw data supporting the conclusions of this article will be made available by the authors, without undue reservation.

## Author contributions

SZ, YY designed the study collected and analysed the data and wrote the manuscript. ZZ analysed the data. LM, CW contributed to collecting the data and discussing the manuscript. MY, XC, TL, RS reviewed and discussed the manuscript. YC designed the study contributed to the data analysis and reviewed and edited the manuscript. All authors have read and approved the final manuscript.

## Funding

This work was supported by research project grants from National Natural Science Foundation of China (81600601) and Natural Science Foundation of Shandong Province(ZR2021MH363).

## Acknowledgments

The authors would like to thank all the reviewers who participated in the review, as well as MJEditor (www.mjeditor.com) for providing English editing services during the preparation of this manuscript.

## Conflict of interest

The authors declare that the research was conducted in the absence of any commercial or financial relationships that could be construed as a potential conflict of interest.

## Publisher’s note

All claims expressed in this article are solely those of the authors and do not necessarily represent those of their affiliated organizations, or those of the publisher, the editors and the reviewers. Any product that may be evaluated in this article, or claim that may be made by its manufacturer, is not guaranteed or endorsed by the publisher.
