# Supplementary material for: Association of obesity, triglyceride-glucose and its derivatives index with risk of hyperuricemia among college students in Qingdao, China
Source: Front Endocrinol (Lausanne). 2022 Oct 6;13:1001844. doi: 10.3389/fendo.2022.1001844 (PMC9583912; doi:10.3389/fendo.2022.1001844)
Supplement: Supplementary file 1 [file DataSheet_1.docx]

| Supplementary form S1,S2 | |  |  |
| --- | --- | --- | --- |
| Variable（Males） | | AUC (95% CI) | P |
| TyG-WC 0.694（0.684，0.704） | BMI | 0.677（0.667，0.687） | ＜0.001 |
|  | WC | 0.678（0.668，0.688） | ＜0.001 |
|  | WHtR | 0.671（0.661，0.681） | ＜0.001 |
|  | TyG index | 0.629（0.618，0.639） | ＜0.001 |
|  | LAP index | 0.682（0.672，0.692） | ＜0.001 |
|  | ABSI | 0.564（0.554，0.575） | ＜0.001 |
|  | BRI | 0.671（0.661，0.681） | ＜0.001 |
|  | TyG-BMI | 0.692（0.683，0.702） | 0.444 |
|  | TyG-WHtR | 0.688（0.678，0.698） | ＜0.001 |
|  | TyG-LAP | 0.683（0.673，0.693） | ＜0.001 |

| Variable（Females） | | AUC (95% CI) | P |
| --- | --- | --- | --- |
| TyG-BMI 0.702（0.685，0.719） | BMI | 0.690（0.673，0.707） | ＜0.001 |
|  | WC | 0.683（0.666，0.701） | 0.001 |
|  | WHtR | 0.681（0.663，0.698） | ＜0.001 |
|  | TyG index | 0.627（0.610，0.644） | ＜0.001 |
|  | LAP index | 0.690（0.673，0.707） | 0.010 |
|  | ABSI | 0.527（0.510，0.545） | ＜0.001 |
|  | BRI | 0.681（0.663，0.698） | ＜0.001 |
|  | TyG-WC | 0.696（0.679，0.713） | 0.155 |
|  | TyG-WHtR | 0.694（0.677，0.711） | 0.071 |
|  | TyG-LAP | 0.689（0.672，0.706） | 0.008 |

Supplementary form S3,S4

| Multivariate logistic regression of different indices for HUA (males). | | |
| --- | --- | --- |
| Variables | Omnibus Tests | Hosmer and Lemeshow Test |
| TyG index |  |  |
| Model 1 | ＜0.001 | 1.000 |
| Model 2 | ＜0.001 | 0.205 |
| Model 3 | ＜0.001 | 0.723 |
|  |  |  |
| BMI |  |  |
| Model 1 | ＜0.001 | 1.000 |
| Model 2 | ＜0.001 | 0.030 |
| Model 3 | ＜0.001 | 0.142 |
|  |  |  |
| WC |  |  |
| Model 1 | ＜0.001 | 1.000 |
| Model 2 | ＜0.001 | 0.030 |
| Model 3 | ＜0.001 | 0.062 |
|  |  |  |
| WHtR |  |  |
| Model 1 | ＜0.001 | 1.000 |
| Model 2 | ＜0.001 | 0.097 |
| Model 3 | ＜0.001 | 0.112 |
|  |  |  |
| LAP index |  |  |
| Model 1 | ＜0.001 | 1.000 |
| Model 2 | ＜0.001 | 0.190 |
| Model 3 | ＜0.001 | 0.129 |
|  |  |  |
| ABSI |  |  |
| Model 1 | ＜0.001 | 1.000 |
| Model 2 | ＜0.001 | 0.029 |
| Model 3 | ＜0.001 | 0.001 |
|  |  |  |
| BRI |  |  |
| Model 1 | ＜0.001 | 1.000 |
| Model 2 | ＜0.001 | 0.058 |
| Model 3 | ＜0.001 | 0.110 |
|  |  |  |
| TyG-BMI |  |  |
| Model 1 | ＜0.001 | 1.000 |
| Model 2 | ＜0.001 | 0.007 |
| Model 3 | ＜0.001 | 0.041 |
|  |  |  |
| TyG-WC |  |  |
| Model 1 | ＜0.001 | 1.000 |
| Model 2 | ＜0.001 | 0.541 |
| Model 3 | ＜0.001 | 0.197 |
|  |  |  |
| TyG-WHtR |  |  |
| Model 1 | ＜0.001 | 1.000 |
| Model 2 | ＜0.001 | 0.321 |
| Model 3 | ＜0.001 | 0.372 |
|  |  |  |
| TyG-LAP |  |  |
| Model 1 | ＜0.001 | 1.000 |
| Model 2 | ＜0.001 | 0.181 |
| Model 3 | ＜0.001 | 0.090 |
| Model 1: unadjusted; model 2: adjusted for age, SBP, and DBP; model 3: adjusted for all variables in model 2 plus BUN, Cre, ALT，AST, and TC. TyG index, triglyceride glucose index; BMI,body mass index; WC, waist circumference; WHtR, waist-to-height ratio; AIP, atherogenic index of plasma; CMI, cardiometabolic index; VAI, visceral adiposity index; LAP index, lipid accumulation product index; ABSI, a body shape index; BRI, body roundness index. | | |

| Multivariate logistic regression of different indices for HUA (females). | | |
| --- | --- | --- |
| Variables | Omnibus Tests | Hosmer and Lemeshow Test |
| TyG index |  |  |
| Model 1 | ＜0.001 | 1.000 |
| Model 2 | ＜0.001 | 0.370 |
| Model 3 | ＜0.001 | 0.975 |
|  |  |  |
| BMI |  |  |
| Model 1 | ＜0.001 | 1.000 |
| Model 2 | ＜0.001 | 0.040 |
| Model 3 | ＜0.001 | 0.478 |
|  |  |  |
| WC |  |  |
| Model 1 | ＜0.001 | 1.000 |
| Model 2 | ＜0.001 | 0.233 |
| Model 3 | ＜0.001 | 0.944 |
|  |  |  |
| WHtR |  |  |
| Model 1 | ＜0.001 | 1.000 |
| Model 2 | ＜0.001 | 0.161 |
| Model 3 | ＜0.001 | 0.900 |
|  |  |  |
| LAP index |  |  |
| Model 1 | ＜0.001 | 1.000 |
| Model 2 | ＜0.001 | 0.561 |
| Model 3 | ＜0.001 | 0.559 |
|  |  |  |
| ABSI |  |  |
| Model 1 | 0.002 | 1.000 |
| Model 2 | ＜0.001 | 0.379 |
| Model 3 | ＜0.001 | 0.166 |
|  |  |  |
| BRI |  |  |
| Model 1 | ＜0.001 | 1.000 |
| Model 2 | ＜0.001 | 0.180 |
| Model 3 | ＜0.001 | 0.912 |
|  |  |  |
| TyG-BMI |  |  |
| Model 1 | ＜0.001 | 1.000 |
| Model 2 | ＜0.001 | 1.000 |
| Model 3 | ＜0.001 | 0.414 |
|  |  |  |
| TyG-WC |  |  |
| Model 1 | ＜0.001 | 1.000 |
| Model 2 | ＜0.001 | 0.177 |
| Model 3 | ＜0.001 | 0.722 |
|  |  |  |
| TyG-WHtR |  |  |
| Model 1 | ＜0.001 | 1.000 |
| Model 2 | ＜0.001 | 0.235 |
| Model 3 | ＜0.001 | 0.309 |
|  |  |  |
| TyG-LAP |  |  |
| Model 1 | ＜0.001 | 1.000 |
| Model 2 | ＜0.001 | 0.602 |
| Model 3 | ＜0.001 | 0.479 |
| Model 1: unadjusted; model 2: adjusted for age, SBP, and DBP; model 3: adjusted for all variables in model 2 plus BUN, Cre, ALT、AST、TC, and LDL-C. TyG index, triglyceride glucose index; BMI,body mass index; WC, waist circumference; WHtR, waist-to-height ratio; AIP, atherogenic index of plasma; CMI, cardiometabolic index; VAI, visceral adiposity index; LAP index, lipid accumulation product index; ABSI, a body shape index; BRI, body roundness index. | | |
